# Reply to Ghaffari et al. “In regard to Cuccia et al.: impact of hydrogel peri-rectal spacer insertion on prostate gland intra-fraction motion during 1.5 T MR-guided stereotactic body radiotherapy.”

**DOI:** 10.1186/s13014-020-01659-4

**Published:** 2020-09-09

**Authors:** Francesco Cuccia, Filippo Alongi

**Affiliations:** 1 Advanced Radiation Oncology Department, Sacro Cuore Don Calabria Hospital, Negrar di Valpolicella, Verona, Italy; 2 University of Brescia, Brescia, Italy

We kindly thank the letter by Ghaffari et al. [1] for their interesting observations concerning our recent study about the potential impact of hydrogel spacer on prostate intrafraction motion during 1.5 T MR-guided stereotactic body radiotherapy [2].

As we have already highlighted in the Discussion of our study, we agree with the Authors about the favorable effect of hydrogel spacer in terms of rectal wall sparing, while the potential impact in terms of organ motion still remains a matter of debate with currently available conflicting evidence [3–5].

As hypothesized by the Authors, a potential role of hydrogel spacer in limiting prostate motion may be related to the squeezing effect on the gland towards the pubic bone, which may also reflect on the theoretical impact on antero-posterior shifts. Conversely, the potential inflammatory reaction provided by the insertion of the gel was not observed in our cohort of patients, who well tolerated the procedure with no relevant aftermaths.

Nonetheless, our analysis confirmed a dosimetric advantage provided by the use of hydrogel spacer in terms of rectal dose exposure, also detecting a positive impact on rotational antero-posterior prostate shifts. Notably, in both the cohorts of patients, the comparison between the pre- and post-MRI sequences, that were considered as surrogates of any potential prostate displacement during the beam-on time, revealed median shift values within the PTV margins, with only a statistically significant difference in the A-P rotational direction. As this experience refers to the first 20 patients treated with MR-guided SBRT, no tolerance value was first set at the beginning of the study, but, with the exception of the A-P rotational shift, the magnitudes of displacement in all the other directions were comparable between the spacer and no-spacer subgroups.

However, a further cineMRI-based analysis of real-time organ motion in a larger sample cohort is currently ongoing in our Department, also with the aim to assess organs at risk motion during the beam-on phase.

Still, we reiterate that these preliminary data, despite being encouraging, need further support provided by long-term follow-up results both in terms of toxicity and larger samples of sessions. Whether the dosimetric advantage and the mitigation of prostate motion leads to a significant clinical benefit needs to be clarified. Consequently, although MR-guided radiotherapy has the potential to deeply refine the target volume delineation process and increase accuracy during the delivery phase [6], a margin reduction strategy would be currently unwise as organ motion uncertainty might be affected by several concurrent factors, as also recommended by other Authors [7].

## Data Availability

Not applicable.

## Abbreviations

MRI: Magnetic resonance imaging
PTV: Planning target volume
SBRT: Stereotactic body radiotherapy
